# Identification of Antiviral Compounds against Monkeypox Virus Profilin-like Protein A42R from *Plantago lanceolata*

**DOI:** 10.3390/molecules27227718

**Published:** 2022-11-09

**Authors:** Leena H. Bajrai, Azzah S. Alharbi, Mai M. El-Day, Abrar G. Bafaraj, Vivek Dhar Dwivedi, Esam I. Azhar

**Affiliations:** 1 Biochemistry Department, Faculty of Sciences, King Abdulaziz University, Jeddah 21362, Saudi Arabia; 2 Special Infectious Agents Unit—BSL3, King Fahd Medical Research Center, King Abdulaziz University, Jeddah 21362, Saudi Arabia; 3 Department of Medical Microbiology and Parasitology, Faculty of Medicine, King Abdulaziz University, Jeddah 21362, Saudi Arabia; 4 Department of Medical Laboratory Sciences, Faculty of Applied Medical Sciences, King Abdulaziz University, Jeddah 21362, Saudi Arabia; 5 Makkah Regional Lab, Ministry of Health, Makkah 21955, Saudi Arabia; 6 Bioinformatics Research Division, Quanta Calculus, Greater Noida 201310, India

**Keywords:** monkeypox, A42R profilin-like protein, antivirals, *Plantago lanceolate*

## Abstract

Infections caused by the monkeypox virus (MPXV) have continued to be transmitted significantly in recent years. However, understanding the transmission mechanism, risk factors, and consequences of infection are still limited. Structure-based drug design for MPXV is at an early stage due to the availability of protein structures that have been determined experimentally. However, the structure of the A42R profilin-like protein of MPXV has been solved and submitted to the structure database. This study illustrated an in silico structure-based approach to identify the potential hit compound against A42R of MPXV. Here, 65 *Plantago lanceolata* compounds were computationally screened against A42R of MPXV. Virtual screening identified top five hits (i) Luteolin 7,3′-Diglucuronide (PubChem ID: 44258091), (ii) Luteolin 7-Glucuronide-3′-Glucoside (PubChem ID: 44258090), (iii) Plantagoside (PubChem ID: 174157), (iv) Narcissoside (PubChem ID: 5481663), and (v) (AlphaE,8S,9R)-N-(3,4-Dihydroxyphenethyl)-8-[(3,4-Dihydroxyphenethyl)Carbamoyl]-9-(1,3-Benzodioxole-5-Yl)-3aalpha,7aalpha-Ethano-1,3-Benzodioxole-5-Acrylamide (PubChem ID: 101131595), with binding energy <−9.0 kcal/mol that was further validated by re-docking and molecular dynamic (MD) simulation. Interaction analysis of re-docked poses confirmed the binding of these top hits to the A42R protein as reported in the reference compound, including active residues ARG114, ARG115, and ARG119. Further, MD simulation and post-simulation analysis support Plantagoside and Narcissoside for substantial stability in the binding pocket of viral protein contributed by hydrogen and hydrophobic interactions. The compounds can be considered for further optimisation and in vitro experimental validation for anti-monkeypox drug development.

## 1. Introduction

Previously, monkeypox cases were limited to African regions, mainly central and west Africa and occasionally to North America. However, 47 human monkeypox cases were also detected in the US Midwest in 2003 [1,2,3]. Recently, global cases of monkeypox have been continuously increasing, including in countries where it was not endemic.

The Monkeypox Virus (MPXV), a zoonotic double-stranded DNA virus similar to the variola major virus (VACV), cowpox virus (CPXV), and vaccinia virus (VACV), causes human monkeypox [4,5,6,7]. MPXV is a member of the genus *Orthopoxvirus* within the *Poxviridae* family. The two distinct genetic clades of the MPXV are the west African and central African (Congo Basin) clades. Here, the latter clade causes more severe disease conditions that are more transmissible [8]. It was first reported in Africa in 1970 in the Democratic Republic of Congo (formerly known as Zaire) [9,10]. However, later in 1997, it was observed that the monkeypox virus (MPXV) strain had genetically changed from the previous existing strain [11]. The native MPXV genome isolated from Zaire (1970), originated from clade 1 and has a 196.8 kb genome with 191 open reading frames (ORFs) [12,13,14]. The strains studied in 1996, 2018, and 2022 showed 191, 190, and 190 ORFs, respectively [15]. In the current outbreak, MPXV originated from clade 3, which is mostly identical to the 2017–2018 strain (with >99% sequence identity) [14,16]. However, the seven proteins from the 1996 strain do not exist in the 2018 and 2022 strains, while both these strains had a similar proteome for all 190 proteins [15]. It is noted that more than 400 genomes of the 2022 strain were submitted to the National Center for Bioinformatic Information (NCBI) GenBank, with a genomic size of 184.7–198.0 kb, consisting of 143–214 open reading frames [17].

Although monkeypox has been reported worldwide, information about this disease is still limited to its transmission, risk factors, clinical representation, and hazardous outcomes. As mentioned by WHO (World Health Organization, Geneva, Switzerland) recently, among the non-endemic regions, the most cases (around 88%) are reported in European regions, 10% in America, 1% in the Eastern Mediterranean regions, and <1% in the Western Pacific regions [18]. In the WHO report, based on the observations captured between 20 and 26 August 2022, there was a surge of 15% of cases globally, where 23% of the increase was in the Americas, 13% in the Western Pacific region, 8% in Southeast Asia, 7% in the European region, and 3% in the Eastern Mediterranean region [18].

MPXV infection is transmitted through large respiratory droplets, salivary secretions, close or direct contact with skin lesions, and possibly through contaminated fomites or items handled by an infected individual [19,20,21]. MPXV performs its replication process in the cytoplasm and its invasion of the host cells consists of three steps: (a) absorption, (b) membrane fusion, and (c) core invasion [22]. After attachment and fusion of the MPXV with the host cell, it starts uncoating and producing early genes during DNA replication, followed by transcription of intermediate and late genes. Detection of MPXV is performed mainly by targeting these genes. There are a variety of methods used to detect MPXV, but RT-PCR is highly preferred. Mostly, the targeted genes for RT-PCR are the DNA polymerase gene (E9L) [23,24], DNA-dependent RNA polymerase subunit 18 genes (RPO18) [25], extracellular envelope protein gene (B6R) and complementary binding proteins 3CL, F3L, and N3R [26,27]. A study on MPXV detection using RT-PCR assay measured the quantification of COP-E9L genes and COP-B5R genes [23].

In addition to labelling proteins and genes for diagnostic purposes, continuous efforts are being made to solve protein structures to guide structure-based drug discovery. However, there is only one experimental protein structure, “A42R profilin-like protein” [17], available in the protein data bank that restricted the structure-based drug discovery [28,29]. A42R is a short protein encoded by the MPXV gp153 locus that demonstrates sequence similarity to eukaryote profilins, actin-binding proteins involved in the regulation of cytoskeletal structure and function [17,30,31]. The study on A42R showed that this protein might have distinct interactions with phosphatidylinositol lipids [17]. In the same study, it was suggested that the role of A42R in the replication of *orthopoxviruses* is not strongly conclusive [17]. However, the results of this study and the availability of the structure propose A42R as a therapeutic target for structure-based drug design.

The study performed in 1970 illustrated the application of the smallpox vaccine against MPXV that could provide 85% protection against MPXV [32,33]. JYNNEOS is the only vaccine approved by the FDA on 9 August 2022, for emergency usage to protect against MPXV [34]. Moreover, the vaccine development and validation programmes are challenging and executed in multiple phases. In addition, immunising a large population with vaccine doses is a time-intensive task. These conditions urgently demand the development of antiviral therapeutics against MPXV that have a relatively shorter development and validation cycle. Antiviral medications could be used on all exposed individuals, even those with underlying immunodeficiency disorders [35]. Currently, to treat monkeypox patients with severe symptoms, Tecovirimat (TPOXX) is administered, which was approved by FDA during the 2022 outbreak [19,36]. Cidofovir and Brincidofovir, approved for smallpox, have also shown activity against MPXV [37]. Cidofovir and Brincidofovir inhibit DNA replication and exhibit inhibitory action against different dsDNA viruses, while Tecovirimat is more specific for *orthopoxviruses* and prevents the formation of the enveloped virus, which is needed for cell-to-cell transmission [38].

There have been several investigations on the antiviral properties of natural compounds sourced from medicinal plants. The traditional medicinal plants from *Plantago* species have been used in modern therapeutic applications due to their astringent, styptic, antimicrobial, expectorant, diuretic, and demulcent characteristics [39,40,41,42]. *Plantago lanceolata,* also known as Ribwort plantain, is an effective wound healer, immunity enhancer, antidiarrheal, skin regenerative, anti-inflammatory, antibacterial, and antiviral agent [43,44,45]. The phenolic profile, antioxidant, anti-inflammatory, and cytotoxic activity of *P. lanceolata* are all higher than its morphologically similar species, which enhanced its efficacy against several ailments [43,46,47]. Drug discovery is increasingly relying on in silico structure-based therapeutic design, which is essential for the identification of cost-effective potential drug molecules. These computational techniques help medicinal chemists and pharmacologists during the drug discovery process by reducing the usage of animal models in pharmacological research, assisting in the rational design of novel and safe drug candidates, and repositioning already-marketed medications [48,49]. In this study, an in silico structure-based drug design approach was demonstrated to detect potential hit compounds from *P. lanceolata* against the A42R protein of MPXV. The current study used the 3D-structure of MPXV-A42R and performed a virtual screening of 65 compounds reported in *P. lanceolata.* Later, molecular dynamic simulation was performed on the top five hits (i) Luteolin 7,3′-Diglucuronide (PubChem ID: 44258091), (ii) Luteolin 7-Glucuronide-3′-Glucoside (PubChem ID: 44258090), (iii) Plantagoside (PubChem ID: 174157), (iv) Narcissoside (PubChem ID: 5481663), and (v) (AlphaE,8S,9R)-N-(3,4-Dihydroxyphenethyl)-8-[(3,4-Dihydroxyphenethyl)Carbamoyl]-9-(1,3-Benzodioxole-5-Yl)-3aalpha,7aalpha-Ethano-1,3-Benzodioxole-5-Acrylamide (PubChem ID: 101131595). Molecular docking and simulation studies showed stable binding characteristics of these compounds at the binding site of the A42R protein.

## 2. Materials and Methods

### 2.1. Structure Collection

The crystal structure of the A42R protein of MPXV Zaire-96-I-16 was sourced from the PDB database [28,29] (PDB ID: 4QWO) [17]. A42R is an actin-binding protein that regulates F-actin assembly. The asymmetric unit of A42R consisted of two polypeptide chains, A and B, solved at a resolution of 1.52 Å composed of 133 amino acids with 24 additional residues at the N-terminal from the tobacco-etch mosaic virus (TEV) protease recognition site. Three alpha helices and one incomplete helix encircled the seven-stranded anti-parallel beta-sheet, which made up the overall structure of A42R. Herein, 3,6,9,12,15,18,21-Heptaoxatricosane-1,23-Diol is a co-crystalised compound (code: PE8) for the A42R protein of MPXV, which was used as a reference. The 3D structure of this native inhibitor was also downloaded from the PubChem database [50] (CID: 78798) [51,52]. Later, 65 *P. lanceolata* compounds were extracted from the PubChem database.

Furthermore, the dock prep programme of the Chimera suite [53] was used to prepare the protein structure, including the addition of polar hydrogens, assignment of the partial charges, and proper bond order allocation under default parameters. Later, the AutoDock [54,55] in the PyRx [56] programme was used in the virtual screening of 65 natural compounds of *P. lanceolata* at the binding site of co-crystallised ligand PE8 of A42R. The ligands, in this case, were created by converting them into the PDBQT format using the built-in OpenBabel tool [57] and minimising their energy using Universal Force Field [58].

### 2.2. Virtual Screening

The binding pocket of the protein was detected with the CASTp server [59]. The grid box dimensions used for virtual screening were 30 Å × 30 Å × 30 Å in x, y, and z directions, covering the binding site residues detected by CASTp that were further verified with the 6 Å neighbouring residues from the reference bound ligand PE8. In virtual screening, the AutoDock programme [54,55] was used to dock each ligand to the specified grid box of A42R. The default settings of the parameters were used for the docking protocol. These were binding modes 10, exhaustiveness 8, and maximum energy difference 3 (kcal/mol). Later, the PyRx results and analysis platform were used to determine the 10 best binding modes for each docking run based on their binding score.

### 2.3. Re-Docking

The top five hits from the virtual screening were re-docked with the A42R protein at the same binding site. The A42R protein and the top five hits were minimised with the help of the structure minimisation tool in the UCSF Chimera using default parameters [53]. The parametrisation of these top five hits and A42R protein was performed using the DockPrep tool in the Chimera with default values. The native ligand was removed, and the polar hydrogen bonds and charges were added to the A42R protein. A grid size of 30 Å × 30 Å × 30 Å in x, y, and z directions, was used with residues centered at 1.90 Å, 4.79 Å, and 19.17 Å, covering the binding site residues. Eventually, these ligands were docked using the Chimera Autodock plugin. Their top docked poses were selected for further molecular dynamic simulation analysis.

### 2.4. Molecular Dynamics (MD) Simulation, PCA Analysis, and ADME

A molecular dynamics (MD) simulation was performed to understand the stability of the docked complexes. The free academic Desmond integrated into Maestro 2020-4 [60] was used to determine the best-scored conformations of the ligand–protein complexes by applying a 100 ns MD simulation. The water box was fixed as (10 Å × 10 Å × 10 Å buffer) for every Protein-ligand complex MD system. Further, the water molecules deploy the TIP4P (transferable intermolecular potential 4 point) model for system minimisation using Desmond–Maestro interface’s System Builder tools. After removal of the salt and ion placement at 20 Å from the ligand, counter ions were added to neutralise the whole system. During MD simulation, the time step was 0.002 ps for the anisotropic diagonal position scaling to retain constant pressure. Further, the temperature was calibrated to 300 K with a 20 ps NPT reassembly at 1 atm pressure for the system while the system density was kept close to 1 g/cm3. All calculations were performed using the default settings. The simulation interaction diagram tool in the free academic Desmond–Maestro program [60] was used to examine the simulation paths. The simulation trajectory was converted into a Bio3D [61,62] compatible format to perform the principal component analysis (PCA) [63]. This application was implemented in the ‘R’ program. Here, the molecule’s initial coordinate was used as a reference, and the other conformations made by the simulation were layered on top of it to find the eigen vector, which represents the orthogonal principal components of the molecule’s motion. The ADME analysis was performed using SwissADME server in order to determine some physicochemical, lipophilicity, water solubility, pharmacokinetics, drug-likeness, and medicinal properties of the molecule [64,65,66,67].

## 3. Results and Discussion

### 3.1. Virtual Screening and Redocking Analysis

Virtual screening was performed for the 65 *P. lanceolata* compounds collected from the PubChem database. This binding site has residues from both chains A and B, which were predicted by the CASTp server [59]. It showed the best pocket with 272.75 Å^2^ surface area and 389.82 Å^3^ volume. The identified pocket consisted of GLU83, TYR118, ARG114, ARG115, ARG119, ARG122, and ASP123 from chain A and the same set of residues from chain B. These binding site residues completely overlapped with the binding location of co-crystalised PE8 molecule in the PDB structure. Table 1 lists the PubChem IDs and binding energies (in kcal/mol) of the 65 compounds in ascending order obtained during the screening.

**Figure 1:**
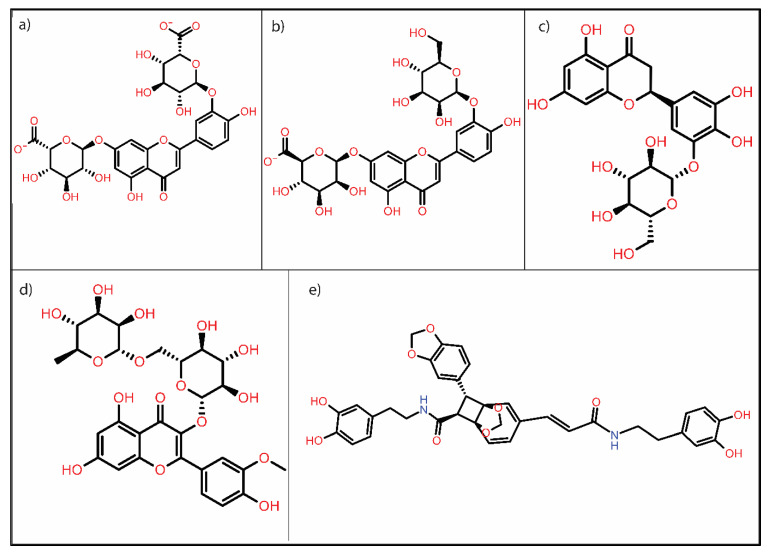
2D structure of top five hits: (**a**) Luteolin 7,3′-diglucuronide, (**b**) Luteolin 7-glucuronide-3′-glucoside, (**c**) Plantagoside, (**d**) Narcissoside, (**e**) (alphaE,8S,9R)-N-(3,4-Dihydroxyphenethyl)-8-[(3,4-dihydroxyphenethyl)carbamoyl]-9-(1,3-benzodioxole-5-yl)-3aalpha,7aalpha-ethano-1,3-benzodioxole-5-acrylamide.

Binding energies calculated by AutoDock were used to rank the compounds, and the top five compounds were selected (shown in bold in Table 1). Here, re-docking was performed with the top five compounds (PubChem IDs: 44258091, 44258090, 174157, 5481663, and 101131595) to confirm their affinities. Figure 1 shows the 2D structures of these top five compounds—(a) Luteolin 7,3′-Diglucuronide (PubChem ID: 44258091), (b) Luteolin 7-Glucuronide-3′-Glucoside (PubChem ID: 44258090), (c) Plantagoside (PubChem ID: 174157), (d) Narcissoside (PubChem ID: 5481663), and (e) (AlphaE, 8S, 9R) -N-(3,4-Dihydroxyphenethyl)-8-[(3,4-Dihydroxyphenethyl) Carbamoyl]-9-(1,3-Benzodioxole-5-Yl)-3aalpha,7aalpha-Ethano-1,3-Benzodioxole-5-Acrylamide (PubChem ID: 101131595). Structure of reference molecule PE8 is shown in Supplementary Materials Figure S1.

### 3.2. Re-Docking Top Hits

**Figure 2:**
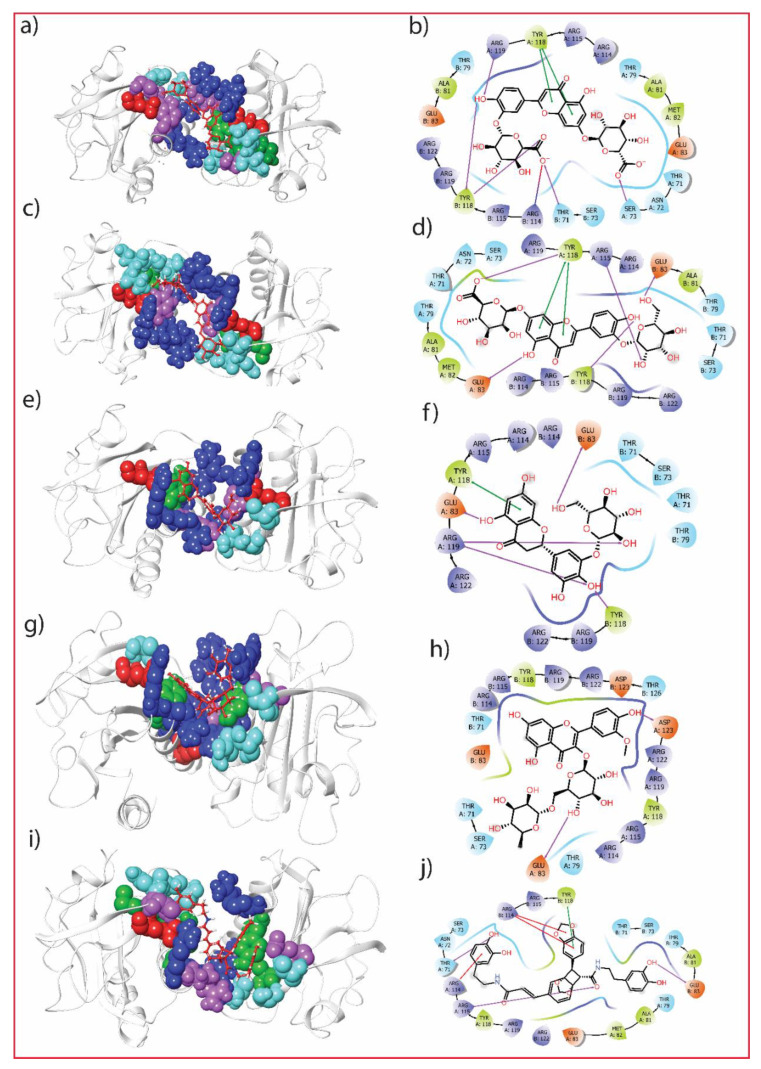
3D and 2D illustration of best poses of the selected top five *P. lanceolata* compounds: (**a**,**b**) Luteolin 7,3′-diglucuronide, (**c**,**d**) Luteolin 7-glucuronide-3′-glucoside, (**e**,**f**) Plantagoside, (**g**,**h**) Narcissoside, and (**i**,**j**) (alphaE,8S,9R)-N-(3,4-Dihydroxyphenethyl)-8-[(3,4-dihydroxyphenethyl)carbamoyl]-9-(1,3-benzodioxole-5-yl)-3aalpha,7aalpha-ethano-1,3-benzodioxole-5-acrylamide.

In Table 1, 65 compounds were ranked according to their binding energies, and the top five were selected for re-docking to reassure their stronger binding affinity for the A42R protein. These top five compounds were further re-docked at the same binding site using AutoDock. In Figure 2, the selected top five compounds showed interactions with binding site residues of the protein A42R for the best pose generated in re-docking. Table 2 shows the binding energies obtained in the re-docking experiment. All the compounds showed the same binding energy score as in virtual screening except for Plantagoside, which differed by 0.1 kcal/mol. The re-docking score confirmed the strong binding of the molecule to the A42R protein. All these hits showed high binding energy <−9.0 kcal/mol. The known co-crystallised ligand, PE8 of the protein A42R, used as a reference compound, was also docked using AutoDock. The reference compound PE8 was docked with the A42R protein, and the best docked score was −4.4 kcal/mol. This showed the binding energies for the top five ligands were better compared to the reference compound PE8.

### 3.3. Protein-Ligand Interaction

The ligand Luteolin 7,3′-diglucuronide (PubChem ID: 44258091) showed the best docking score of −9.9 kcal/mol among all the 65 *P. lanceolata* compounds listed in Table 1. The residues SER73, ARG119 from chain A, and THR71, TYR118 from chain B of the protein A42R showed direct bonding with the ligand Luteolin 7,3′-diglucuronide using hydrogen bonds, as shown in Figure 2b, while its corresponding 3D pose is shown in Figure 2a. The stacking interaction for this molecule was formed with TYR118 of chain A. The compound Luteolin 7-glucuronide-3′-glucoside (PubChem ID: 44258090) showed a binding energy of −9.6 kcal/mol. Here, GLU83, ARG115, Tyr118 of chain A, and Glu83 and Tyr118 of chain B showed hydrogen bond interaction with the ligand Luteolin 7-glucuronide-3′-glucoside, which has been depicted in Figure 2d, and its corresponding 3D pose is shown in Figure 2c. Additionally, this compound had a stacking (ring) interaction with TYR118 of chain B. The binding energy of the compound Plantagoside (PubChem ID: 174157) was −9.0 kcal/mol in re-docking while −9.1 kcal/mol in virtual screening. Chain A residues, Glu83, Arg119 and chain B residues, Glu83, Tyr118 of the protein formed H-bonds with the ligand Plantagoside. This compound also had the same stacking interaction with the ring of TYR118, as shown in Figure 2f, and its corresponding 3D pose is shown in Figure 2e. The compound Narcissoside (PubChem ID: 5481663) and compound alphaE,8S,9R)-N-(3,4-Dihydroxyphenethyl)-8-[(3,4-dihydroxyphenethyl)carbamoyl]-9-(1,3-benzodioxole-5-yl)-3aalpha,7aalpha-ethano-1,3-benzodioxole-5-acrylamide (PubChem ID: 101131595) showed a binding energy of −9.0 kcal/mol in the virtual screening and in re-docking. The protein A42R residues Glu83 and Asp123 from chain A formed hydrogen bonds with the ligand Narcissoside, as shown in Figure 2h, and its corresponding 3D pose is shown in Figure 2g. This molecule did not show any stacking interaction with the protein. The list of interacting residues and type of interactions for A42R protein of MPXV with the top five hits and reference molecule PE8 are listed in Table 3.

Lastly, Thr71, Arg115 of chain A and Glu83 of chain B showed H-bond interactions with the ligand (alphaE,8S,9R)-N-(3,4-Dihydroxyphenethyl)-8-[(3,4-dihydroxyphenethyl)carbamoyl]-9-(1,3-benzodioxole-5-yl)-3aalpha,7aalpha-ethano-1,3-benzodioxole-5-acrylamide, shown in Figure 2j and its corresponding 3D pose is shown in Figure 2i. This compound also formed a stacking interaction with ARG114 and TYR118 from chain A and ARG114 from chain B of the protein. Table 2 summarises the interactions of the A42R protein residues with these selected compounds (H-bond, hydrophobic, polar, π–π stacking/π-cation, and salt bridges positive/negative). It has been observed that all these hits were bound at almost similar locations in the protein binding site as shown in Figure 2a,c,e,g,i. The Supplementary Figure S2 and Table 2 show the binding sites of the A42R protein with the reference ligand PE8, which showed the formation of H-bonds with the GLU77, ASN78 from chain B and ARG127, ARG119 from chain A. Here, ARG119 was the residue that was involved in the hit compound interaction and in the reference compound.

### 3.4. Molecular Dynamics Analysis

**Figure 3:**
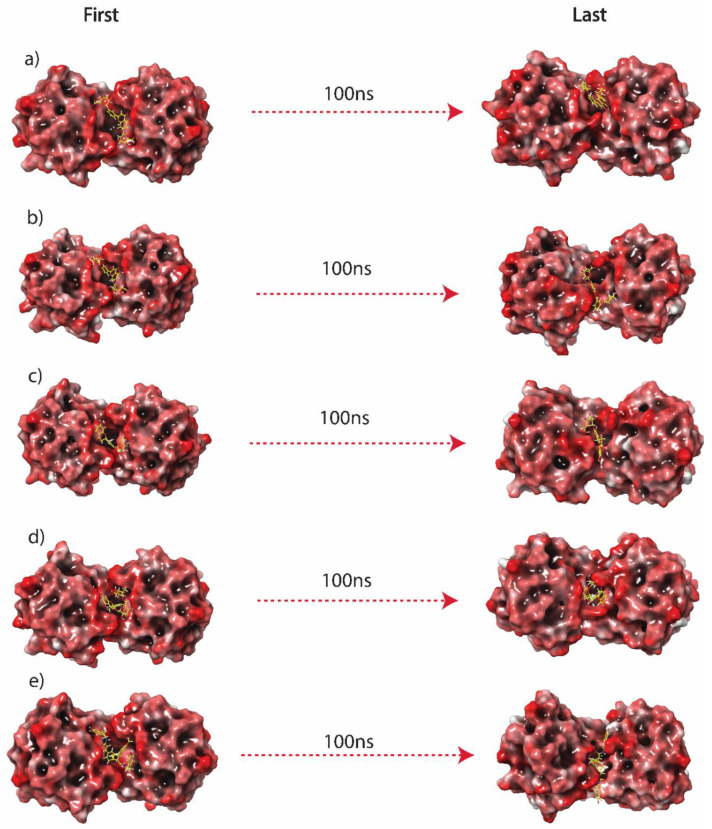
Representation of the 3D surface conformational changes in the last poses, i.e., (**a**) Luteolin 7,3′-diglucuronide, (**b**) Luteolin 7-glucuronide-3′-glucoside, (**c**) Plantagoside, (**d**) Narcissoside, (**e**) (alphaE,8S,9R)-N-(3,4-Dihydroxyphenethyl)-8-[(3,4-dihydroxyphenethyl)carbamoyl]-9-(1,3-benzodioxole-5-yl)-3aalpha,7aalpha-ethano-1,3-benzodioxole-5-acrylamide, extracted from the 100 ns MD simulation trajectories in comparison to the respective docked poses.

The application of molecular dynamics simulation is essential to provide a comprehensive understanding of the dynamics of molecules, including Protein-ligand complexes. Here, a 100 ns explicit solvent molecular dynamic simulation was performed to capture the interactions of MPXV protein A42R with the top five hits screened from virtual screening. The entire system was equilibrated in a solvated box to settle the ions and water molecules before the 100 ns production phase. The conformational changes between the initial and final poses that resulted from the simulation are shown in Figure 3. Here, the final pose of the molecule changed from the initial pose both in rotational and translation space. In Figure 3a Luteolin 7,3′-diglucuronide compound showed significant translation motion within the binding site of the protein. It translated upward in the binding site, which rotated to attain a compact globular structure. Figure 3b shows the initial and final pose of Luteolin 7-glucuronide-3′-glucoside in the complex with A42R. Here, it can be observed that the compound rotated, but the translation motion is smaller than the first compound. Figure 3c represents Plantagoside, showing horizontal translation while a lower magnitude of rotation. The Narcissoside compound is shown in Figure 3d, where there was high conformational consistency, and the final pose resembled the initial pose in translational rotational space. Figure 3e showed the initial and final poses of (alphaE,8S,9R)-N-(3,4-Dihydroxyphenethyl)-8-[(3,4-dihydroxyphenethyl)carbamoyl]-9-(1,3-benzodioxole-5-yl)-3aalpha,7aalpha-ethano-1,3-benzodioxole-5-acrylamide where the compound attained the extended conformation within the binding pocket in final pose compared to initial pose. Similarly, reference compound PE8 was also analysed for the initial and final pose generated in the simulation. Supplementary Figure S3 shows these poses for the reference compound. It can be observed that reference compound PE8 moved to a different location at the end of the simulation in a high magnitude, which was later also confirmed in its RMSD behaviour.

#### 3.4.1. RMSD Analysis

**Figure 4:**
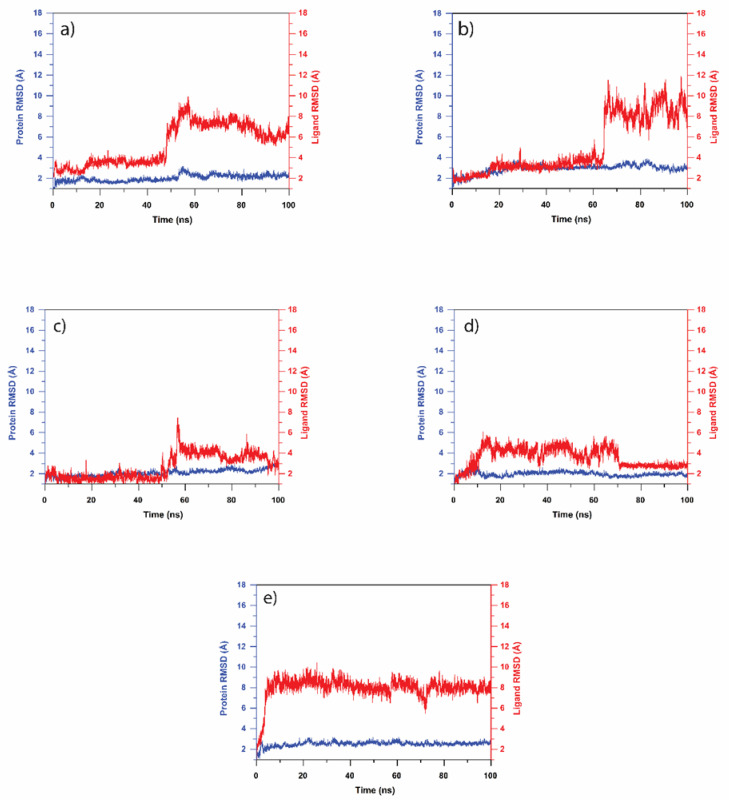
RMSD plots for natural compounds: (**a**) Luteolin 7,3′-diglucuronide, (**b**) Luteolin 7-glucuronide-3′-glucoside, (**c**) Plantagoside, (**d**) Narcissoside, (**e**) (alphaE,8S,9R)-N-(3,4-Dihydroxyphenethyl)-8-[(3,4-dihydroxyphenethyl)carbamoyl]-9-(1,3-benzodioxole-5-yl)-3aalpha,7aalpha-ethano-1,3-benzodioxole-5-acrylamide. (Ligand RMSD is in red, and Protein RMSD is in blue.)

The RMSD (root mean square deviation) analysis in MD simulation provides critical information about the conformational stability of the complexes. This magnitude of RMSD determines the molecule’s deviation from its initial conformation calculated for all frames of the simulation trajectory. In this case, the RMSD was independently estimated for the entire trajectory of the protein and the ligand to provide the conformational variation within the system lower than 3 Å between two states, which indicates no significant or minimal change, which is often acceptable when a molecular comparison is considered. A conformationally consistent molecule has a similar RMSD with the initial conformation, showing a parallel graph plot with the X-axis. Figure 4a–e shows the RMSD of the selected compounds over the 100 ns trajectory. In this study, ligand RMSD values were calculated as the protein-fit for all docked complexes from their respective 100 ns MD simulation trajectories, while the RMSD of the protein was calculated for the Cα atoms. In ligand RMSD, a protein molecule was used as a reference for the fit, and the RMSD of the ligand was computed. This accounts for the translational and rotational motion of the ligand molecule in the RMSD. The relative RMSD was consistently under 3 Å for MPXV protein A42R, demonstrating its conformational stability during the MD simulation. As shown in Figure 4, the RMSD of the protein (indicated with blue lines) was under 2 Å for all the selected top five compound complexes. The RMSD for the compound Luteolin 7-glucuronide-3′-glucoside, on the other hand, reached 3 as shown in Figure 4b. There were various fluctuation patterns that were formed in the ligand RMSD (indicated with red lines), as shown in Figure 4. The ligand Luteolin 7,3′-diglucuronide showed a consistent fluctuation of 3–4 Å during the first 50 ns of simulation and then spiked up to 10, indicating the compound’s low stability in the Protein-ligand complex, as shown in Figure 4a. Running on a similar RMSD trend, the RMSD of the ligand Luteolin 7-glucuronide-3′-glucoside was below 3 Å for the first 20 ns, and it reached to 3–4 Å between 20–65 ns. Figure 4b shows that it eventually spiked to 7.5–12 Å during 65–100 ns, indicating its unstable nature in the ligand–protein complex. Plantagoside, on the other hand, exhibited relatively higher conformational consistency: the ligand RMSD was consistently less than 3 Å during the first 50 ns, then spiked between 4–6 Å during the last 50 ns before returning to 3 Å at 100 ns, as shown in Figure 4c. Supplementary Figure S4a shows the RMSD for the reference compound complex. Here, the protein showed a consistent variation around 3 Å.

The RMSD of the ligand Narcissoside during the simulation showed RMSD under 3 Å during the first 10 ns and eventually increased to 6 Å and fluctuated till 70 ns. However, in the final 30 ns, it dropped to 3 until the end of the 100 ns, as shown in Figure 4d. The compound (alphaE,8S,9R)-N-(3,4-Dihydroxyphenethyl)-8-[(3,4-dihydroxyphenethyl)carbamoyl]-9-(1,3-benzodioxole-5-yl)-3aalpha,7aalpha-ethano-1,3-benzodioxole-5-acrylamide showed an increase in RMSD during the initial 10 ns followed by a state of equilibrium (<8.2 Å) till the end of 100 ns MD simulation, as shown in Figure 4e. Comparing the test compounds, Plantagoside and Narcissoside showed the minimum structural variation, with an RMSD value of under 3 Å in the 50% frames of the simulation, indicating higher stability of the ligand in the given Protein-ligand complex. The reference ligand, PE8, was also tested for RMSD and demonstrated lower stability with a maximum deviation ranging from 5 to 12 Å during the simulation, as shown in Supplementary Figure S4a.

#### 3.4.2. RMSF Analysis

RMSF (root mean square fluctuation) computes the conformational change for every individual residue. Here, the RMSFs were calculated for each residue of A42R during a 100 ns simulation. The amino acids in a protein that contribute the most to its molecular motion can be identified by plotting the RMSF per residue against the number of residues. The Supplementary Figure S5a–e shows RMSF plots for proteins docked with natural compounds computed for the Cα atoms of the residue. In addition, Figure S6 shows RMSF plots for the top compounds that were computed for each atom of the compound. Figure S5 shows the RMSF plot for the combined chains A and B of proteins numbered continuously. In the protein molecule, none of the residues showed RMSF > 3 Å except for a large spike shown for the N-terminal and C-terminal residues for chains A and B. The spike shown in the middle of the plot for each complex represents the C-terminal of chain A and the starting N-terminal of chain B. A similar trend of RMSF is observed in the reference compound complex (Supplementary Figure S4b). In contrast to protein molecules, ligands showed relatively higher RMSF. Most atoms of the ligand had an RMSF > 3 Å. However, Narcissoside had the most stable RMSF pattern (2 Å) for the first 31 atoms, but it jumped >3 Å for the last 10 atoms. Plantagoside also showed lower RMSF (<3 Å) for all its atoms except the 31st atom. Luteolin 7-glucuronide-3′-glucoside compound consistently showed RMSF~4 Å for all its atoms. Reference compound RMSF is shown in Figure S4c; here, the compound had RMSF between 6–8 Å for all the atoms of reference compound.

### 3.5. MD Simulation Protein-Ligand Interaction

**Figure 5:**
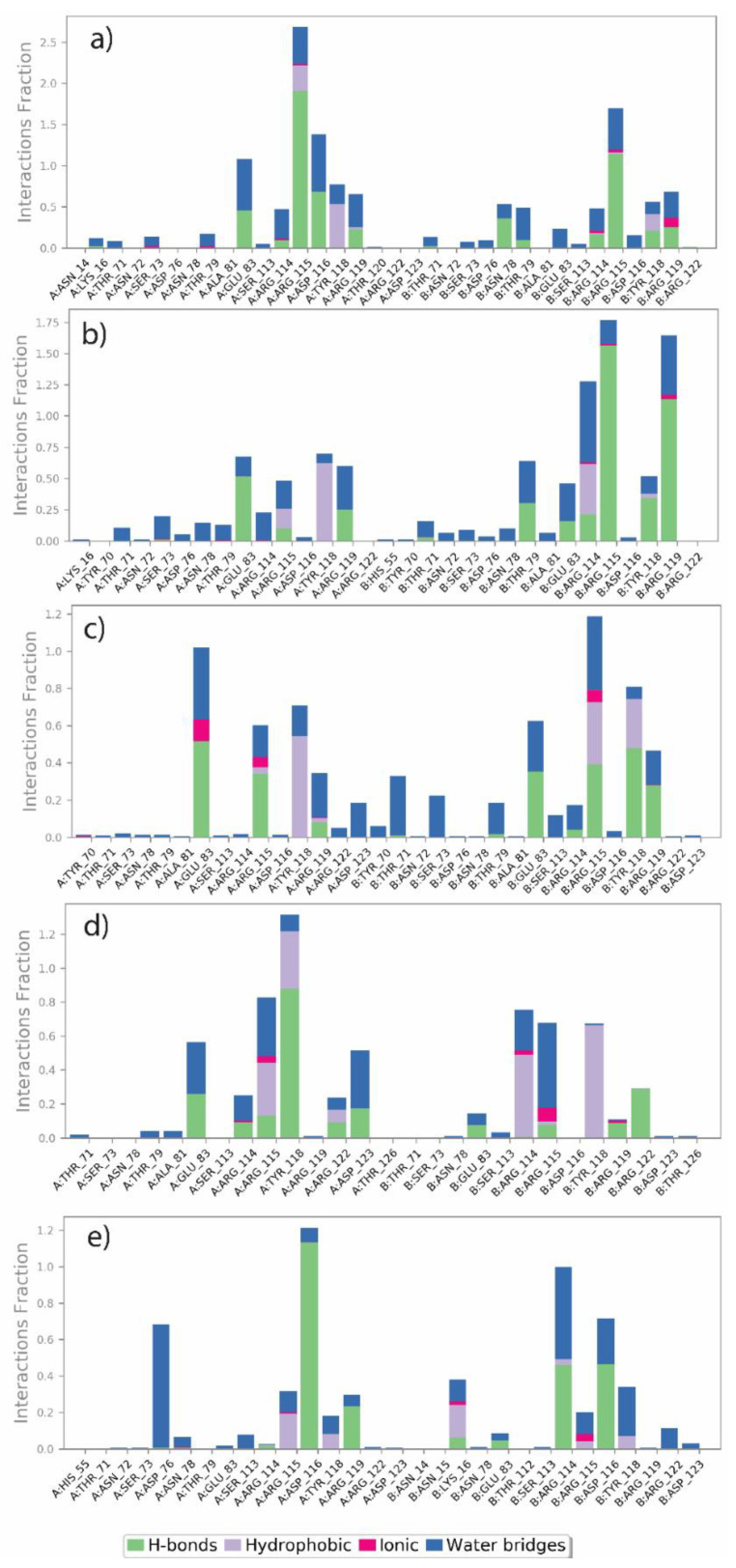
Protein-ligand interactions mapping A42R and selected natural compounds: (**a**) Luteolin 7,3′-diglucuronide, (**b**) Luteolin 7-glucuronide-3′-glucoside, (**c**) Plantagoside, (**d**) Narcissoside, (**e**) (alphaE,8S,9R)-N-(3,4-Dihydroxyphenethyl)-8-[(3,4-dihydroxyphenethyl)carbamoyl]-9-(1,3-benzodioxole-5-yl)-3aalpha,7aalpha-ethano-1,3-benzodioxole-5-acrylamide. Fits on protein were extracted from 100 ns MD simulation trajectories of respective docked complexes.

The data generated from the MD simulation was further used to analyse the atomic level interactions between the selected natural compounds and the protein A42R. These interactions include hydrogen bonds, hydrophobic contacts, ionic interactions, and water bridges. Therefore, these Protein-ligand interactions were studied for the 100 ns simulation trajectories to confirm the complex stability. Figure 5 shows the interaction fraction for the binding site residue of the protein with the respective ligand. Figure 5a shows that ARG115 from chains A and B had the highest H-bond formation fraction in the Luteolin 7,3′-diglucuronide complex. ALA81 and ASP116 from chain A also showed significant H-bond participation across all frames of MD simulation. These residues also play a critical role in water bridge formation. Similarly, ARG115 and ARG119 from chain B contributed predominantly to H-bond formation in Luteolin 7-glucuronide-3′-glucoside. In this molecule, the higher interacting contribution was made by chain B of protein. As shown in Figure 5b, ARG114 (chain B) contributed the most to the formation of water bridges for this molecule. Both these molecules (Luteolin 7,3′-diglucuronide and Luteolin 7-glucuronide-3′-glucoside) have similar scaffolds and show similar residues involved in forming strong interactions. In contrast to these molecules, Plantagoside involved GLU83 (chain A) and TYR118 (chain B) as the most critical contributors to H-bonds. However, ARG115 (chain A) and ARG119 (chain B) also showed H-bonds in a significant number of MD simulation frames, as shown in Figure 5c. Here, THR71 (chain B) and THR73 (chain B) are predominantly involved in water bridges. In Narcissoside, the fraction of H-bond is lower compared to the above three compounds. However, as shown in Figure 5d, TYR118 (chain A) had a higher H-bond fraction, whereas ARG115 (chain B) was involved in water bridge formation. Lastly, (alphaE,8S,9R)-N-(3,4-Dihydroxyphenethyl)-8-[(3,4-dihydroxyphenethyl)carbamoyl]-9-(1,3-benzodioxole-5-yl)-3aalpha,7aalpha-ethano-1,3-benzodioxole-5-acrylamide compound showed a similar interaction profile as Luteolin 7,3′-diglucuronide and ARG115 from both chains majorly responsible for the H-bond, while GLU83 (chain A) and ASP116 (chain B) are the two most critical residues involved in water bridges. Supplementary Figure S6 shows the interaction map of these ligands with proteins with the percentage and type of interaction they formed with protein residues. All the top-hit compounds interacted more strongly than the reference PE8 (shown in Supplementary Figure S7). Interestingly, it was observed from Figure 5 that the residues ARG115 and ARG119 from chain B and TYR118, and GLU83 from chain A of the protein A42R interacted with all the selected natural compounds in a large number of frames. However, the reference did not show any interaction with these residues. Herein, the reference showed the most interaction with ARG127 and ARG129 of chains A and B, respectively, of the targeted protein A42R. Figure 5 and Figure S7 show that the selected compounds had more significant intermolecular interaction than the reference. Considering the above analysis, it suggested that selected natural compounds of *P. lanceolata* were more stable than the reference PE8 during the 100 ns molecular dynamics simulation.

### 3.6. Principal Component Analysis (PCA) and ADME

**Figure 6:**
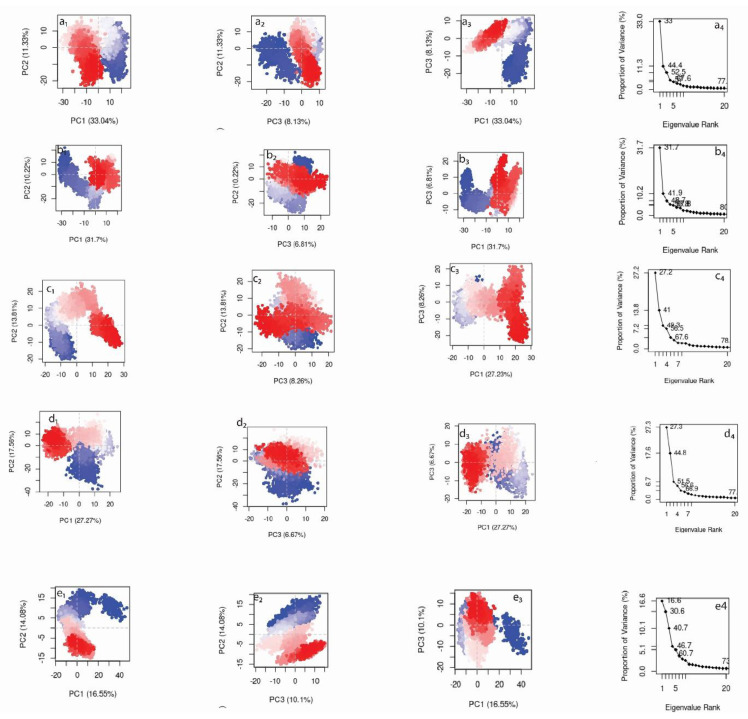
Principal component analysis (PCA) for the top three principal components obtained by dimensionality reduction of the motion observed in the 100 ns MD simulation. (**a_1_**–**a_4_**) Luteolin 7,3′-diglucuronide, (**b_1_**–**b_4_**) Luteolin 7-glucuronide-3′-glucoside, (**c_1_**–**c_4_**) Plantagoside, (**d_1_**–**d_4_**) Narcissoside, (**e_1_**–**e_4_**) (alphaE,8S,9R)-N-(3,4-Dihydroxyphenethyl)-8-[(3,4-dihydroxyphenethyl)carbamoyl]-9-(1,3-benzo-dioxole-5-yl)-3aalpha,7aalpha-ethano-1,3-benzodioxole-5-acrylamide.

PCA in this study was performed to estimate the conformational motion of the protein after binding to the top five hits. PCA for the reference molecule was also accessed for the comparative analysis. A protein molecule (A42R) exhibits high dimensional motion in the system during the simulation. PCA breaks down dimensions into primary essential components. Here, the first three principal components were examined for each protein-ligand complex. These are considered statistically significant conformational motions of the protein shown during the MD simulation. Figure 6 shows the PCA analysis for the complexes of the top five compounds, while Supplementary Figure S8 Z plots the top three principal compounds for the reference complex. Figure 6 shows that the first three principal components accounted for 52.5%, 48.7%, 49.3%, 51.5%, and 40.7%, respectively, of the total conformational variation for the top five hits. The top three principal components were plotted in pairs to show conformational variation with the progress of simulation. These conformational motions were shown in different colours, where blue and red represent the variational degree, while the gradient from blue to red via white shows the time steps in simulation. Luteolin 7,3′-diglucuronide clustered in the initial time step of the simulation at PC1 +20 (blue) while it transformed and reached another cluster at PC1 −10 (red). Compared with the Luteolin 7-glucuronide-3′-glucoside, conformational transition trend is different from that of Luteolin 7,3′-diglucuronide. However, on PC1 and PC2 scales, protein molecules in both the complexes showed similar dispersion behaviour. Plantagoside showed a relatively smoother transition (blue–white–red) compared to the first two complexes. Moreover, this transition on the principal component (PC1) scale was non-overlapping. However, in Narcissoside, these transitional conformations are overlapping on PC1, as clusters in different colours had the same PC1 value. Moreover, it also reflects the minimum structural variation of the protein in the conformation space. Lastly, (alphaE,8S,9R)-N-(3,4-Dihydroxyphenethyl)-8-[(3,4-dihydroxyphenethyl)carbamoyl]-9-(1,3-benzodioxole-5-yl)-3aalpha,7aalpha-ethano-1,3-benzodioxole-5-acrylamide showed the most dispersed characteristics on the PC2 scale, while on the PC1 scale there were overlapping conformational clusters found at the initial and final time step of the MD simulation. PCA for the reference is shown in Supplementary Figure S8. Here, the top three principal components constituted 45% of the total motion, where the plot between PC1-PC2 showed a periodic conformational transformation from one state to another state. The ADME results of all the five compounds are given in Supplementary Table S1.

## 4. Conclusions

This study started with the in silico detection of A42R binding site using the CASTp online tool. It was found that the binding site detected in this study substantially overlapped with the binding location of the PE8 molecule that co-crystalised with A42R of MPXV. In addition, phosphatidylinositol-(4,5)-bisphosphate (PIP2), which is a natural substrate that A42R also binds at a similar location in MPXV. Further, this study screened 65 *Plantago lanceolata* compounds against the detected binding site of the protein. Initial phase of virtual screening resulted in the identification of top five hit compounds that were further evaluated in a re-docking experiment. All these compounds had significantly negative binding energies (−9.0 kcal/mol), indicating their significant binding affinity. Moreover, the residue interactions shown in the best docked poses confirmed their interaction with the functional residues that bind with PIP2 (Arg114, Arg115, and Arg119). Later, the flexibility of the binding behaviour was determined using 100 ns MD simulation. When the RMSD of these compounds was calculated in their complex form for the whole simulation trajectory, Plantagoside and Narcissoside showed a very stable pattern compared to the other compounds (RMSD 3–5). This strongly advocated the Protein-ligand complex stability for these two compounds. Other compounds showed translational motion that was reflected by their RMSD jump of >8 Å. However, they also retained the functional residue interactions. Overall, this study reported that five compounds sourced from *Plantago lanceolata* had a high potential to bind at the active site of A42R of MPXV and inhibit natural substrate binding.

## Figures and Tables

**Table 1:** Binding energies were calculated using Pyrx for 65 *P. lanceolata* compounds screened against the A42R protein of MXV. Top five hits are highlighted.

Ligand (PubChem ID)	Binding Energy (kcal/mol)	Ligand (PubChem ID)	Binding Energy (kcal/mol)	Ligand (PubChem ID)	Binding Energy (kcal/mol)
44258091	−9.9	5281778	−8.4	5280443	−7.3
44258090	−9.6	5281788	−8.4	91458	−7.1
174157	−9.1	5318083	−8.4	5280863	−7.1
5481663	−9	10494	−8.2	5319292	−7.1
101131595	−9	14034195	−8.1	91520	−6.9
5280805	−9	5315651	−8.1	100332	−6.6
44593361	−9	24892726	−8.1	14132338	−6.4
5280601	−8.9	84298	−7.9	689043	−6.2
9986606	−8.9	44258433	−7.9	12300213	−6.2
5280637	−8.8	5281605	−7.8	132560907	−6.1
5319484	−8.8	5318987	−7.8	445858	−6.1
6476333	−8.8	1794427	−7.7	637775	−5.9
21603201	−8.7	64945	−7.7	72	−5.7
154809630	−8.6	107848	−7.7	370	−5.7
442664	−8.6	5280445	−7.6	3469	−5.6
5280704	−8.6	46173850	−7.6	1549106	−5.6
5320623	−8.6	5280343	−7.5	8468	−5.6
44423103	−8.6	5281672	−7.5	444539	−5.5
6476333	−8.5	9064	−7.4	135	−5.4
5273567	−8.5	443354	−7.4	338	−5.3
5281800	−8.5	11968737	−7.4	−−	−−
5280804	−8.4	5280633	−7.3	−−	−−

**Table 2:** Re-docking score of top five hits selected from the virtual screening.

Compounds	Re-Docking Score (kcal/mol)
Luteolin 7,3′-diglucuronide (44258091)	−9.9
Luteolin 7-glucuronide-3′-glucoside (44258090)	−9.6
Plantagoside (174157)	−9.0
Narcissoside (5481663)	−9.0
(alphaE,8S,9R)-N-(3,4-Dihydroxyphenethyl)-8-[(3,4-dihydroxyphenethyl)carbamoyl]-9-(1,3-benzodioxole-5-yl)-3aalpha,7aalpha-ethano-1,3-benzodioxole-5-acrylamide (101131595)	−9.0
**PE8 (Reference)**	−4.4

**Table 3:** List of interacting residues and type of interactions for A42R protein of MPXV with the top five hits and reference molecule PE8.

S.No.	Complex	H-Bond	Hydrophobic	Polar	π–π Stacking/π-Cation	Positive	Negative
**1**	Monkey pox—Luteolin 7,3′-diglucuronide	A: Ser^73^, A: Arg^119^, B: Thr^71^, B: Tyr^118^	A: Ala^81^, A: Met^82^, A: Tyr^118^, B: Ala^81^, B: Tyr^118^	A: Thr^71^, A:Asn^72^, A:Ser^73^, A: Thr^79,^ B: Thr^71^, B: Ser^73^, B:Thr^79^	A: Tyr^118^	A: Arg^114^ A: Arg^115^ A: Arg^119^ B: Arg^114^ B: Arg^115^ B: Arg^119^ B: Arg^122^	A: Glu^83^ B: Glu^83^
**2**	Monkey pox—Luteolin 7-glucuronide-3′-glucoside	A: Glu^83^ A: Arg^115^ A: Tyr^118^ B: Glu^83^ B: Tyr^118^	A: Ala^81^, A: Met^82^, A: Tyr^118^, B: Ala^81^, B: Tyr^118^	A: Thr^71^, A:Asn^72^, A: Ser^73^, A: Thr^79^, B: Thr^71^, B: Ser^73^, B:Thr^79^	A: Tyr^118^	A: Arg^114^ A: Arg^115^ A: Arg^119^ B: Arg^114^ B: Arg^115^ B: Arg^119^ B: Arg^122^	A: Glu^83^ B: Glu^83^
**3**	Monkey pox—Plantagoside	A: Glu^83^ A: Arg^119^ B: Glu^83^ B: Tyr^118^	A: Tyr^118^ B: Tyr^118^	A: Thr^71^ B: Thr^71^, B: Ser^73^, B:Thr^79^	A: Tyr^118^	A: Arg^114^ A: Arg^115^ A: Arg^119^ A: Arg^122^ B: Arg^114^ B: Arg^115^ B: Arg^119^ B: Arg^122^	A: Glu^83^ B: Glu^83^
**4**	Monkey pox—Narcissoside	A: Glu^83^ A: Asp^123^	A: Tyr^118^ B: Tyr^118^	A: Thr^71^, A: Ser^73^, A: Thr^79^, B: Thr^71^ B: Thr^126^	--	A: Arg^114^ A: Arg^115^ A: Arg^119^ A: Arg^122^ B: Arg^114^ B: Arg^115^ B: Arg^119^ B: Arg^122^	A: Glu^83^ A: Asp^123^ B: Glu^83^ B: Asp^123^
**5**	Monkey pox—(alphaE,8S,9R)-N-(3,4-Dihydroxyphenethyl)-8-[(3,4-dihydroxyphenethyl)carbamoyl]-9-(1,3-benzodioxole-5-yl)-3aalpha,7aalpha-ethano-1,3-benzodioxole-5-acrylamide	A: Thr^71^, A: Arg^115^ B: Glu^83^	A: Ala^81^, A: Met^82^, A: Tyr^118^, B: Ala^81^, B: Tyr^118^	A: Thr^71^, A:Asn^72^, A: Ser^73^, A: Thr^79^, B: Thr^71^, B: Ser^73^, B:Thr^79^	A: Tyr^118^ A: Arg^114^ * B: Arg^114^ *	A: Arg^114^ A: Arg^115^ A: Arg^119^ A: Arg^122^ B: Arg^114^ B: Arg^115^	A: Glu^83^ B: Glu^83^
**6**	PE8 (reference)	A:Arg^127^, A: Arg^119^, B: Glu^77^, B: Asn^78^	A: Ile^11^, A: Phe^17^, B: Tyr^80^	A: Asn^14^, A: Thr^120^, A: His^124^, B: Asn^78^, B: Thr^126^	--	A: Lys^16^, A: Arg^115^, A: Arg^119^, A: Arg^127^, B: Arg^129^, B: Arg^122^,	A: Asp^10^, A: Asp^116^, A: Asp^123^, B: Glu^78^

Symbol asterisk (*) symbol represents the residues showing * _π-Cation interactions.

## Data Availability

The datasets used and/or analyzed during the current study are already given the manuscript, and the raw data generated during analysis are available from the corresponding author on reasonable request.

## Supplementary Materials

The following supporting information can be downloaded at: https://www.mdpi.com/article/10.3390/molecules27227718/s1, Figure S1; Reference ligand structure; Figure S2; Target-reference ligand interaction; Figure S3, First and last pose of reference molecule; Figure S4; RMSD and RMSF plots of reference compound in complex with the target protein; Figure S5; RMSF plots for protein docked with natural compounds; Figure S6; Ligand protein contacts of all the selected compounds after MD simulations; Figure S7; Protein-ligand contact mapping for reference ligand after MD simulation; Figure S8; PCA analysis of reference compound in complex with the target protein; Table S1; ADME analysis of all the selected compounds.
