# Supplementary material for: Identification of Antiviral Compounds against Monkeypox Virus Profilin-like Protein A42R from Plantago lanceolata
Source: Molecules. 2022 Nov 9;27(22):7718. doi: 10.3390/molecules27227718 (PMC9697570; doi:10.3390/molecules27227718)
Supplement: Supplementary file 1 [file molecules-27-07718-s001.zip › molecules-1966319-supplementary.pdf]

Identification of antiviral compounds against Monkeypox virus profilin-like protein A42R from *Plantago lanceolata*

Leena H. Bajrai[1,2*], Azzah S. Alharbi[2,3], Mai M. El-Day[2,4], Abrar G. Bafaraj[2,5], Vivek Dhar Dwivedi[2,6*] and Esam I. Azhar[2,4*]

[1] Biochemistry Department, Faculty of Sciences, King Abdulaziz University, Jeddah 21362, Saudi Arabia
[2] Special Infectious Agents Unit—BSL3, King Fahd Medical Research Center, King Abdulaziz University, Jeddah 21362, Saudi Arabia
[3] Department of Medical Microbiology and Parasitology, Faculty of Medicine, King Abdulaziz University, Jeddah 21362, Saudi Arabia
[4] Department of Medical Laboratory Sciences, Faculty of Applied Medical Sciences, King Abdulaziz University, Jeddah 21362, Saudi Arabia
[5] Makkah Regional Lab, Ministry of Health, Makkah, Saudi Arabia
[6] Bioinformatics Research Division, Quanta Calculus, Pvt. Ltd., Greater Noida 201310, India
* Correspondence: lbajrai@kau.edu.sa; vivek_bioinformatics@yahoo.com; eazhar@kau.edu.sa

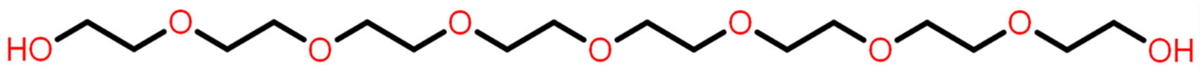

Figure S1- control 3,6,9,12,15,18,21-HEPTAOXATRICOSANE-1,23-DIOL

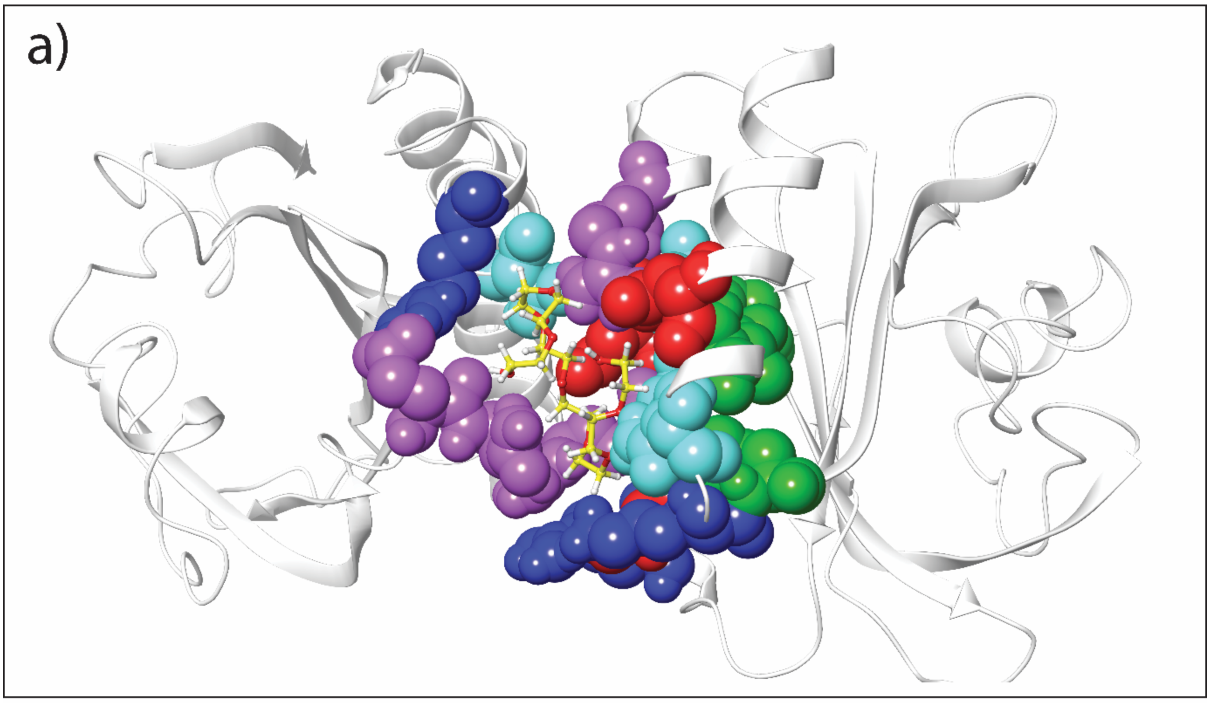

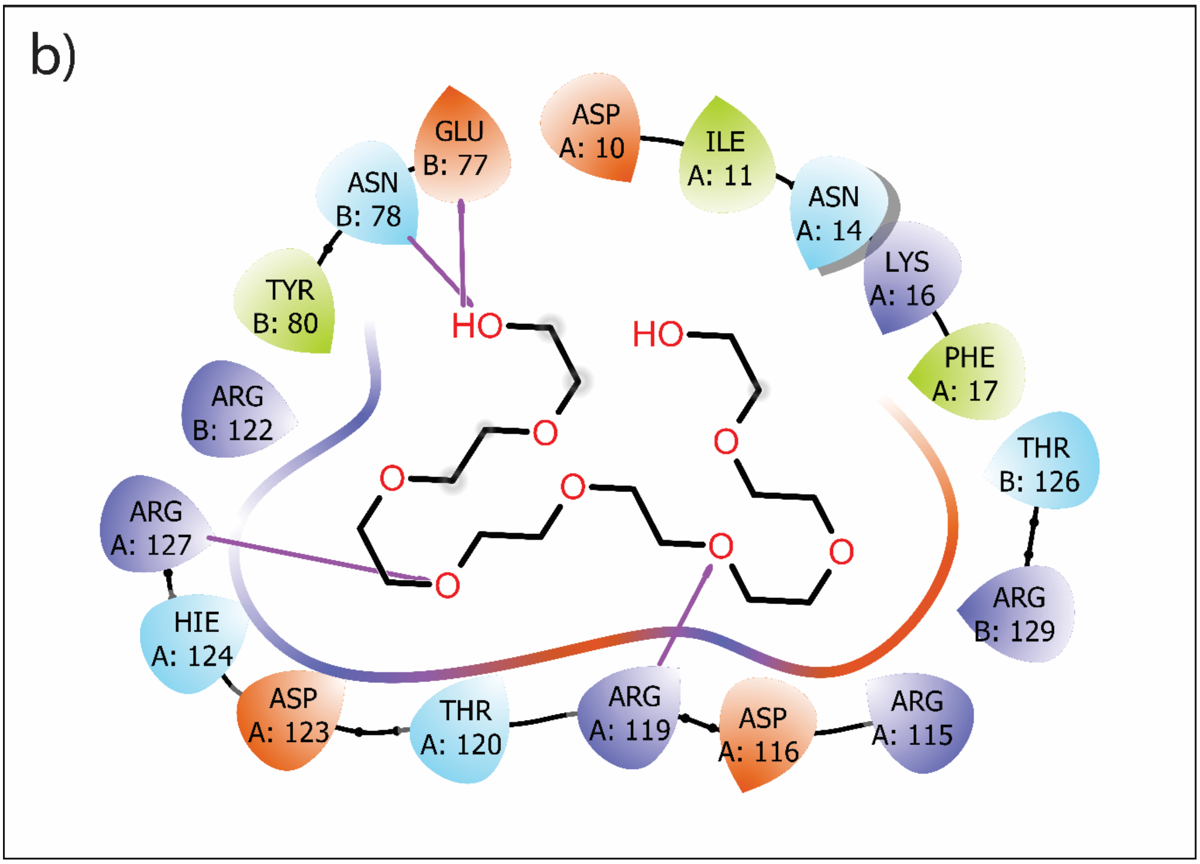

Figure S2- 2d and 3d pose of control

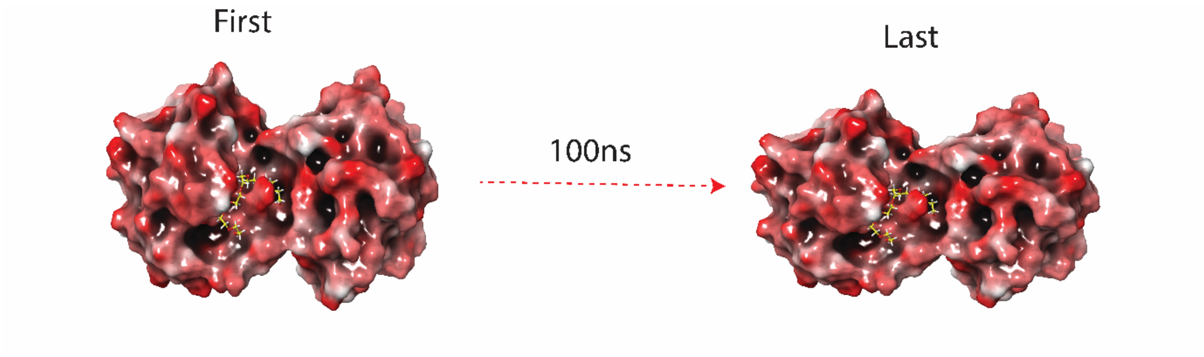

Figure S3 – first and last pose of control

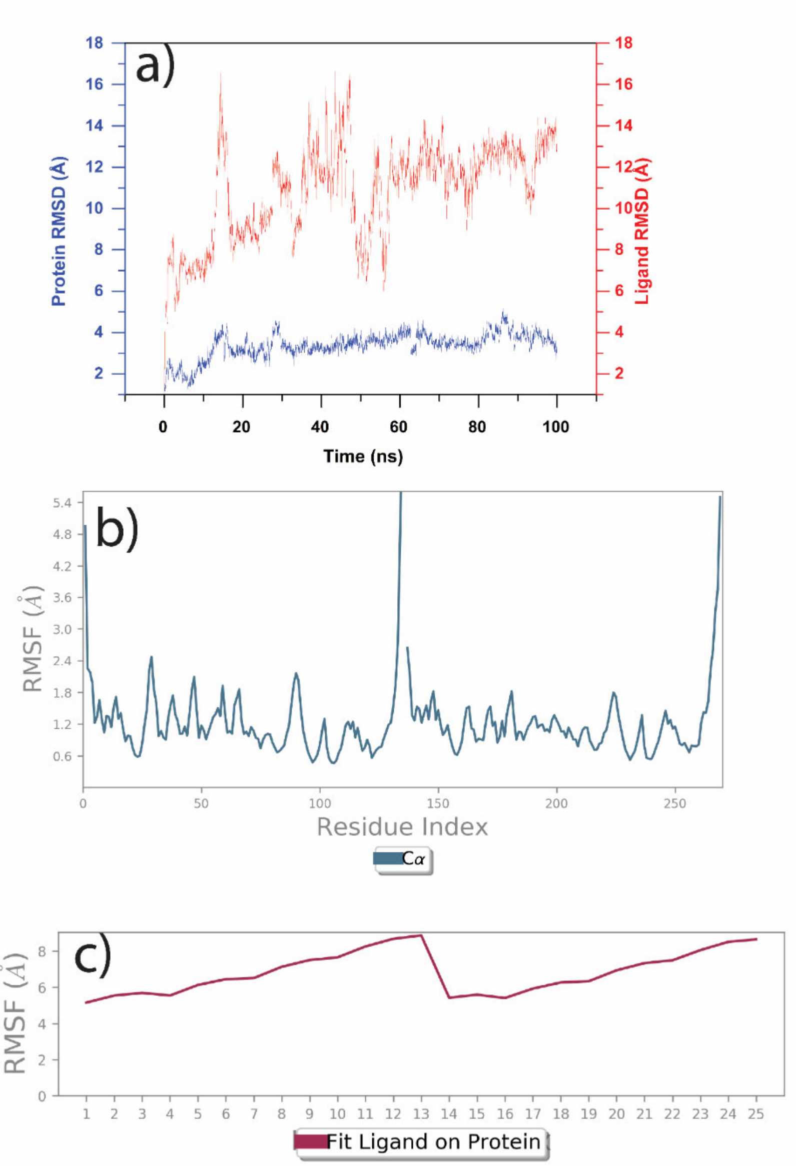

Figure S4- a) RMSD of control b) RMSF plot of protein- control c) RMSF Plot of ligand-control

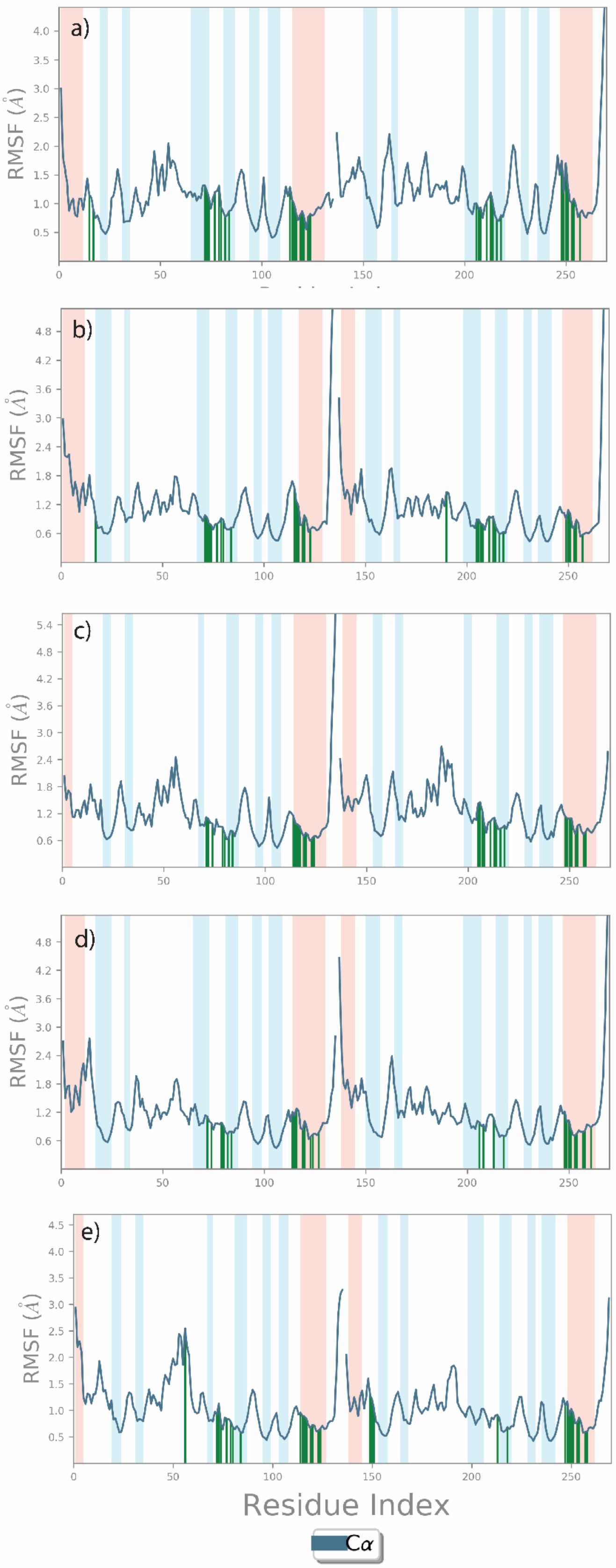

Figure S5. RMSF plots for protein docked with natural compounds, i.e., a) Luteolin 7,3'-diglucuronide b) Luteolin 7-glucuronide-3'-glucoside c) Plantagoside d) Narcissoside e) (alphaE,8S,9R)-N-(3,4-Dihydroxyphenethyl)-8-[(3,4-dihydroxyphenethyl)carbamoyl]-9-(1,3-

benzodioxole-5-yl)-3a alpha,7a alpha-ethano-1,3-benzodioxole-5-acrylamide as function of 100 ns simulation interval.

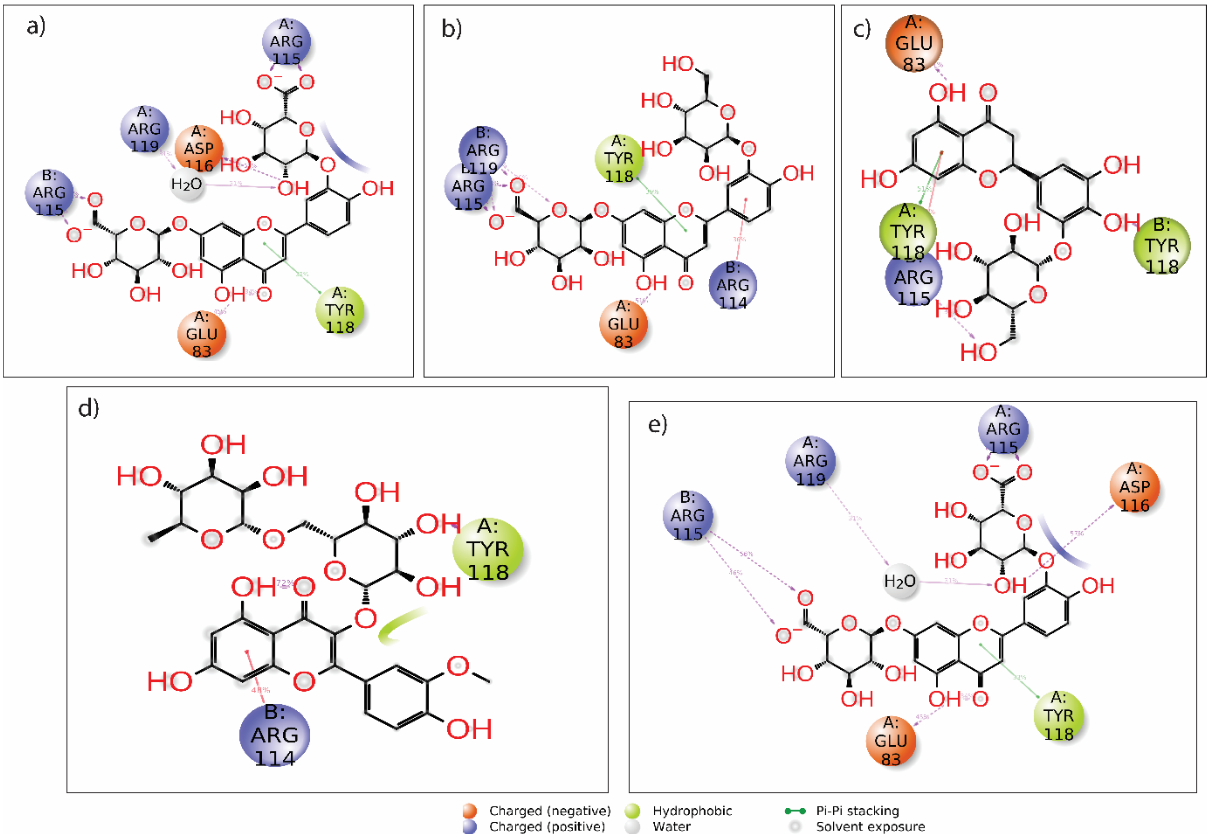

Figure S6. Ligand protein contact of PE8 and selected natural compounds, i.e., (a) Luteolin 7,3'-diglucuronide b) Luteolin 7-glucuronide-3'-glucoside c) Plantagoside d) Narcissoside e) (alphaE,8S,9R)-N-(3,4-Dihydroxyphenethyl)-8-[(3,4-dihydroxyphenethyl)carbamoyl]-9-(1,3-benzodioxole-5-yl)-3aalpha,7aalpha-ethano-1,3-benzodioxole-5-acrylamide fit on protein were extracted from 100 ns MD simulation trajectories of respective docked complexes.

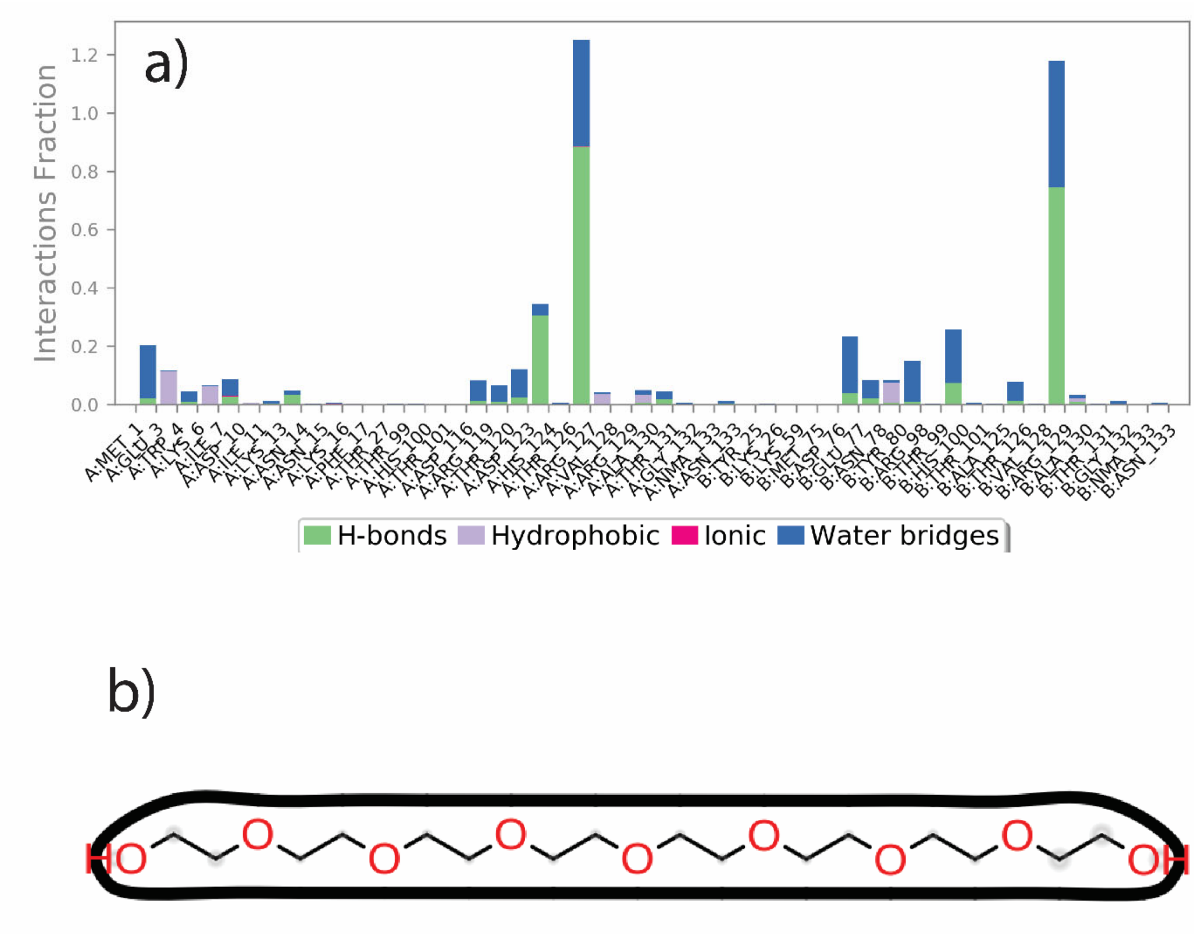

Figure S7 a) protein-ligand interaction of control b) protein-ligand contact of control

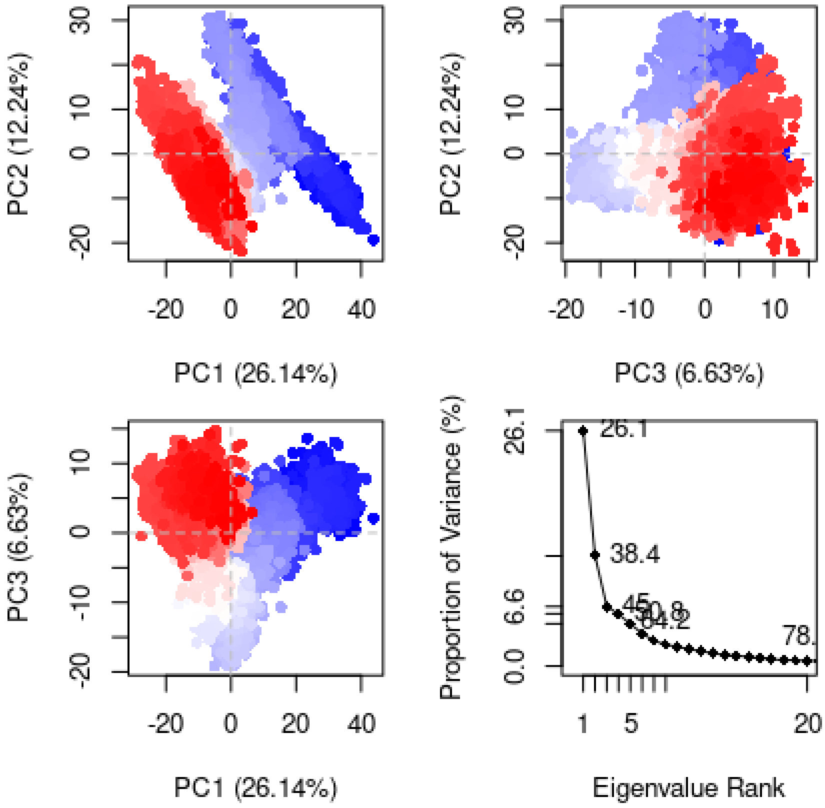

Figure S8. PCA of control

Table S1. ADME analysis of all the selected compounds.

**Molecule**	**44258091**	**44258090**	**174157**	**5481663**	**101131595**
Formula	C27H26O18	C27H28O17	C21H22O12	C28H32O16	C36H34N2O10
MW	638.48	624.5	466.39	624.54	654.66
#Heavy atoms	45	44	33	44	48
#Aromatic heavy atoms	16	16	12	16	18
Fraction Csp3	0.37	0.41	0.38	0.46	0.28
#Rotatable bonds	7	7	4	7	12
#H-bond acceptors	18	17	12	16	10
#H-bond donors	10	10	8	9	6
MR	141.48	140.87	107.74	145.85	171.93
TPSA	303.57	286.5	206.6	258.43	176.04
iLOGP	1.32	1.16	1.65	2.62	3.58
XLOGP3	0.15	-0.1	-0.06	-0.01	2.9
WLOGP	-2.59	-2.68	-0.93	-1.38	3.2
MLOGP	-4.13	-4.15	-2.4	-3.69	1.26
Silicos-IT Log P	-3.12	-2.68	-1.06	-1.55	3.8
Consensus Log P	-1.67	-1.69	-0.56	-0.8	2.95
ESOL Log S	-3.69	-3.46	-2.7	-3.51	-5.21
ESOL Solubility (mg/ml)	0.129	2.19E-01	9.33E-01	1.92E-01	4.02E-03
ESOL Solubility (mol/l)	0.000202	3.50E-04	2.00E-03	3.07E-04	6.15E-06
ESOL Class	Soluble	Soluble	Soluble	Soluble	Moderately soluble
Ali Log S	-6.08	-5.46	-3.83	-4.97	-6.26
Ali Solubility (mg/ml)	0.000529	2.15E-03	6.94E-02	6.73E-03	3.62E-04
Ali Solubility (mol/l)	0.000000828	3.44E-06	1.49E-04	1.08E-05	5.53E-07
Ali Class	Poorly soluble	Moderately soluble	Soluble	Moderately soluble	Poorly soluble
Silicos-IT LogSw	0.65	0.17	-0.52	-0.97	-6.99
Silicos-IT Solubility (mg/ml)	2820	9.22E+02	1.40E+02	6.66E+01	6.75E-05
Silicos-IT Solubility (mol/l)	4.42	1.48E+00	2.99E-01	1.07E-01	1.03E-07
Silicos-IT class	Soluble	Soluble	Soluble	Soluble	Poorly soluble
GI absorption	Low	Low	Low	Low	Low
BBB permeant	No	No	No	No	No
Pgp substrate	Yes	Yes	No	Yes	No
CYP1A2 inhibitor	No	No	No	No	No
CYP2C19 inhibitor	No	No	No	No	No
CYP2C9 inhibitor	No	No	No	No	No
CYP2D6 inhibitor	No	No	No	No	No
CYP3A4 inhibitor	No	No	No	No	No

log Kp (cm/s)	-10.09	-10.18	-9.19	-10.12	-8.23
Lipinski #violations	3	3	2	3	3
Ghose #violations	4	4	1	4	3
Veber #violations	1	1	1	1	2
Egan #violations	1	1	1	1	1
Muegge #violations	4	4	3	4	3
Bioavailability Score	0.11	0.11	0.17	0.17	0.17
PAINS #alerts	0	0	1	0	1
Brenk #alerts	0	0	1	0	2
Leadlikeness #violations	1	1	1	1	2
Synthetic Accessibility	6.29	6.33	5.14	6.64	6.91
